# Supplementary material for: Dynamic Implant Surgery—An Accurate Alternative to Stereolithographic Guides—Systematic Review and Meta-Analysis
Source: Dent J (Basel). 2023 Jun 8;11(6):150. doi: 10.3390/dj11060150 (PMC10297201; doi:10.3390/dj11060150)
Supplement: Supplementary file 1 [file dentistry-11-00150-s001.zip › dentistry-2279456-supplementary.pdf]

Supplementary Material

Supplementary Table S1. Quality assessment of the cohort studies via the Newcastle–Ottawa Scale (NOS).

	Selection				Comparability	Outcome			
**Author, year**	Representativeness of the exposed cohort	Selection of the non-exposed cohort	Ascertainment of exposure	Demonstration that the outcome of interest was not present at the start of the study	On the basis of the design or analysis controlled for confounders	Assessment of outcomes	Follow-up was long enough for outcomes to occur	Adequacy of following up cohorts	**Score**
**Block et al. [21] (2017)**	*	*	*	*	**	*	*	*	9/9
Dong Wu et al. [46] 2020	*	*	*	*	*	*	*	*	8/9
Ting-Mao Sun et al. [35] 2020	*	*	*	*	*	*	*	*	8/9

Supplemental Table S2. Quality assessment of the randomized clinical trials via the Cochrane scale.

Author, year	Random sequence generator	Allocation concealment	Blinding of participants and personnel	Blinding of assessment of the outcomes	Incomplete outcome data	Selective reporting	Other sources of bias	Overall risk of bias
Farley et al. [36] 2013	Moderate	Low	Low	Low	Low	Low	Moderate	Moderate
Pei Shen et al. [50] 2015	Moderate	Low	Low	Low	Low	Low	Low	Moderate
Faris Younes et al. [41] 2018	Low	Low	Low	Low	Low	Low	Low	Low
Keskanya Subbalekha et al. [42] 2019	Low	Low	Low	Low	Moderate	Low	Low	Moderate
Magrin et al. [43]2019	Low	Low	Low	Low	Low	Low	Low	Low
Palita Smitkarn et al. [45] 2019	Low	Low	Low	Low	Low	Low	Low	Low
Aktolun Aydemir et al. [37] 2020	Low	Low	Low	Low	Low	Low	Moderate	Moderate
Paweena Yimarj et al. [47] 2020	Moderate	Low	Low	Low	Low	Low	Low	Moderate

Supplementary Table S3. Quality assessment of the case series via the Robins-I Scale.

Study	*Before the intervention*	During the intervention	*After the intervention*	Overall risk of bias				Overall risk of bias
	**Bias due to confounding**	*Bias in the selection of participants in the study*	**Bias in classification of the intervention**	**Bias due to deviation from the intended interventions**	**Bias due to missing data**	**Bias in measurement of the outcomes**	**Bias in selection of the reported results**	
Elian et al. [51] (2008)	Moderate	Moderate	Low	Low	Low	Low	Low	Moderate
Ahmet Ersan Ersoy et al. [40] 2008	Low	Low	Low	Low	Low	Low	Low	Low
Oguz Ozan et al. [52] 2009	Modderate	Moderate	Low	Low	Low	Low	Low	Moderate
Van Assche et al. [53] 2010	Moderate	Moderate	Low	Low	Low	Low	Low	Moderate
Platzer et al. [54] 2011	Moderate	Moderate	Low	Low	Low	Low	Low	Moderate
Fürhauser et al. [55] 2014	Low	Low	Low	Low	Low	Low	Low	Low
Du-Hyeong Lee et al. [39] 2016	Moderate	Moderate	Low	Low	Low	Low	Low	Moderate
Schnutenhaus et al. [34](2016)	Low	Low	Low	Low	Low	Low	Low	Low
Naziri et al. [33] 2016	Low	Low	Low	Low	Low	Low	Low	Low
Pellegrino et al. [44]2017	Moderate	Moderate	Low	Low	Low	Low	Low	Moderate
Stefanelli et al. [48]2020	Low	Low	Low	Low	Low	Low	Low	Low
Stefanelli et al. [49] 2020	Low	Low	Low	Low	Low	Low	Low	Low
Stefanelli et al. [38] (2020)	Low	Low	Low	Low	Low	Low	Low	Low
